# Ceramide kinase regulates TNF-α-induced immune responses in human monocytic cells

**DOI:** 10.1038/s41598-021-87795-7

**Published:** 2021-04-15

**Authors:** Fatema Al-Rashed, Zunair Ahmad, Ashley J. Snider, Reeby Thomas, Shihab Kochumon, Motasem Melhem, Sardar Sindhu, Lina M. Obeid, Fahd Al-Mulla, Yusuf A. Hannun, Rasheed Ahmad

**Affiliations:** 1 Immunology & Microbiology Department, Dasman Diabetes Institute, Al-Soor Street, Dasman, P.O. Box 1180, 15462 Kuwait, Kuwait; 2 Royal College of Surgeons in Ireland, Busaiteen, Bahrain; 3 Stony Brook Cancer Center and Department of Medicine, Stony Brook University, Stony Brook, NY 11794 USA; 4 Department of Nutritional Sciences, College of Agriculture and Life Sciences, University of Arizona, Tucson, AZ 85721 USA; 5 Genetics and Bioinformatics Department, Dasman Diabetes Institute, Kuwait, Kuwait; 6 Animal and Imaging Core Facility, Dasman Diabetes Institute, Kuwait, Kuwait

**Keywords:** Immunology, Biomarkers

## Abstract

Ceramide kinase (CERK) phosphorylates ceramide to produce ceramide-1-phosphate (C1P), which is involved in the development of metabolic inflammation. TNF-α modulates inflammatory responses in monocytes associated with various inflammatory disorders; however, the underlying mechanisms remain not fully understood. Here, we investigated the role of CERK in TNF-α-induced inflammatory responses in monocytes. Our results show that disruption of CERK activity in monocytes, either by chemical inhibitor NVP-231 or by small interfering RNA (siRNA), results in the defective expression of inflammatory markers including CD11c, CD11b and HLA-DR in response to TNF-α. Our data show that TNF-α upregulates ceramide phosphorylation. Inhibition of CERK in monocytes significantly reduced the secretion of IL-1β and MCP-1. Similar results were observed in CERK-downregulated cells. TNF-α-induced phosphorylation of JNK, p38 and NF-κB was reduced by inhibition of CERK. Additionally, NF-κB/AP-1 activity was suppressed by the inhibition of CERK. Clinically, obese individuals had higher levels of CERK expression in PBMCs compared to lean individuals, which correlated with their TNF-α levels. Taken together, these results suggest that CERK plays a key role in regulating inflammatory responses in human monocytes during TNF-α stimulation. CERK may be a relevant target for developing novel therapies for chronic inflammatory diseases.

## Introduction

Ceramide kinase (CERK) is an enzyme that regulates phosphorylation of ceramide and produces ceramide-1-phosphate (C1P), a sphingolipid which is commonly implicated in inflammation^1^. CERK is the only enzyme identified so far to induce the biosynthesis of C1P in mammalian cells and this enzyme was first detected in brain synaptic vesicles^2^. CERK is found to be present in both the microsomal membrane fraction and in the cytosolic fraction of cells^3^. It is speculated that different cell types may have different subcellular distribution and expression levels of CERK. The enzyme activity is regulated by Ca^++^ ions as well as phosphorylation/dephosphorylation processes; while, its location and activity require the integrity of the PH domain including a myristoylation site^4^. Involvement of CERK in inflammation is well documented^5,6^. It was reported that the deficiency of CERK suppressed inflammation in adipose tissue through attenuating MCP-1/CCR2 signaling in infiltrated macrophages and improved insulin resistance, pinpointing CERK as a potential therapeutic target for the treatment of obesity and insulin resistance^7^. Furthermore, the CERK- knockout (KO) mice expressed lower levels of MCP-1 which is a well-known inflammatory marker in the adipose tissue. Moreover, MCP-1-induced infiltration of macrophages into the adipose tissue was significantly reduced in CERK KO mice, which associated with reduced diet-induced obesity and metabolic inflammation, as well as with increased insulin sensitivity in these mice^7^.

Obesity and metabolic syndrome are characterized by chronic low-grade inflammation, mainly originating from the crosstalk between monocytes/macrophages and adipocytes in the adipose tissue. In obesity settings, monocytes exhibit an inflammatory phenotype associated with the increased expression of CD11b, CD11c and HLA-DR surface markers^8,9^, along with higher secretion of proinflammatory cytokines/chemokines such as TNF-α, IL-6, IL-1β, and MCP-1^8,10^. TNF-α is a proinflammatory cytokine overexpressed in obese humans and rodents and it has been identified as a key regulator of inflammation and insulin resistance^11^. TNF-α activates the immune cells, particularly monocytes and macrophages into a pro-inflammatory state^8^. However, the underlying mechanisms are still not clear. Since CERK is involved in metabolic inflammation, we investigated whether CERK regulated the phenotypic changes in monocytes stimulated with TNF-α. We found that the inhibition of CERK by either a specific chemical inhibitor or by siRNA significantly blocked the TNF-α-induced expression of inflammatory monocyte markers including CD11b, CD11c and HLA-DR. In parallel, inhibition of CERK suppresses the TNF-α-mediated secretion of inflammatory cytokine/chemokine, such as IL-1β/MCP-1, respectively. Mechanistically, CERK inhibition decreased the TNF-α-induced phosphorylation of JNK, p38 and NF-κB. As expected, we also found the increased expression of CERK in PBMCs of obese individuals which correlated with TNF-α expression. Altogether, our data show an interesting novel role of CERK in TNF-α-driven inflammation.

## Materials and methods

All methods were performed in accordance with the relevant guidelines and regulations.

### Cell culture

Human monocytic THP-1 cells were purchased from American Type Culture Collection (ATCC) and grown in RPMI-1640 culture medium (Gibco, Life Technologies, Grand Island, USA) supplemented with 10% fetal bovine serum (Gibco, Life Technologies, Grand Island, NY, USA), 2 mM glutamine (Gibco, Invitrogen, Grand Island, NY, USA), 1 mM sodium pyruvate, 10 mM HEPES, 100 μg/ml Normocin, 50 U/ml penicillin and 50 μg/ml streptomycin (P/S; (Gibco, Invitrogen, Grand Island, NY, USA). Cells were then incubated at 37 °C (with humidity) in 5% CO_2_. NF-κB/AP-1 reporter monocytic cells (THP-1-XBlue cells) stably expressing a secreted embryonic alkaline phosphatase (SEAP) reporter, inducible by NF-κB and AP-1, were purchased from InvivoGen (InvivoGen, San Diego, CA, USA). THP-1-XBlue cells were cultured in complete RPMI medium with the addition of zeocin (200 μg/ml) (InvivoGen, San Diego, CA, USA). Prior to stimulation, monocytes were transferred to normal medium and plated in 12-well plates (Costar, Corning Incorporated, Corning, NY, USA) at 1 × 10^6^ cells/well cell density unless indicated otherwise.

### PBMCs collection and monocyte purification

Human peripheral blood (30 ml) samples were collected from healthy volunteers in EDTA vacutainer tubes. All participants gave written informed consent and the study was approved by the ethics committee of Dasman Diabetes Institute, Kuwait (Ref.# 04/07/2010; RA-2010-003). Physical characteristics of the study participants are shown in s Table 1. PBMCs were isolated by using Histo-Paque density gradient method as described^12^. PBMCs were plated in 6-well plates (Costar, Corning Incorporated, Corning, NY, USA) at 3 × 10^6^ cells/well cell density and cultured in starvation medium for 3 h at 37 °C. Non-adhered cells were removed, and monocytes adhered to the plate were washed with culture medium without serum and later incubated for 24 h in RPMI with 2% fetal bovine serum.

**Table 1:** Descriptive characteristics of the study population.

Physical characteristics of subjects	Lean (N = 9)	Overweight (N = 8)	Obese (N = 10)	*p-*value
Age (years)	39.2 ± 11.1	42.6 ± 14.0	46.1 ± 12.3	0.4744
Weight (kg)	60.1 ± 8.25	74.4 ± 9.88	104 ± 17	< 0.0001****
Height (cm)	1.64 ± 0.06	1.62 ± 0.12	1.70 ± 0.09	0.1724
BMI (kg/m^2^)	22.1 ± 2.4	28.05 ± 1.05	35.5 ± 3.6	< 0.0001****
Waist circumference (in.)	78 ± 3.6	93.5 ± 3.6	115.27 ± 13.9	0.0012**
Hip circumference (in.)	95.3 ± 4.6	107.5 ± 5.91	114.6 ± 6.8	0.0025**
Fat weight (kg)	30.2 ± 5.9	37.12 ± 3.92	38.3 ± 4.2	0.0663
Lean weight (kg)	35.8 ± 2.6	42.4 ± 5.3	58.8 ± 12.1	0.0071**

p-values represent the difference between lean and obese population.

**** Highly Significance; ** Very significance

### Cell stimulation

Monocytes were plated in 12-well plates (Costar, Corning Incorporated, Corning, NY, USA) at 1 × 10^6^ cells/well concentration unless indicated otherwise. Cells were pre-treated with potent, selective and reversible CERK inhibitor NVP-231 (Tocris, 12 nM) for one hour, then stimulated with TNF-α (10 ng/ml) or Vehicle (0.1% BSA) for 2 h at 37 °C. Cells were harvested for RNA isolation. To assess cytokines secretion (IL-β and MCP-1) in culture media, TNF-α stimulation was carried out for 12 h. For MAPKs and NF-κkB signaling pathway analysis, cultures were treated with the inhibitor as stated above, then stimulated with TNF-α or BSA (vehicle) for 10–15 min.

### Lipidomics

For lipid extraction, cells were washed with ice-cold PBS, then directly lysed with 2 ml cell extraction mixture (2:3 70% isopropanol/ethyl acetate), followed by gentle scraping of cells from the culture plate. The lysate was transferred to 15 ml Falcon tubes. The lipid samples were spiked with C17-sphingosine (C17-SPH) and C17-dihydrosphingosine (C17-DHS) (internal standards, 50 pmol), and extracts were then analyzed by the Lipidomics Core Facility at Stony Brook University Medical Center, as described previously^13^. Data were normalized by total lipid phosphate (Pi) present in the organic phase of the Bligh and Dyer extraction^14^ and detected by phosphomolybdate assay^15^. Sphingolipid levels were expressed as pmol/nmol Pi.

### Real time quantitative RT-PCR

Total RNA was extracted using RNeasy Mini Kit (Qiagen, Valencia. CA, USA) per the manufacturer’s instructions. The cDNA was synthesized using 1 μg of total RNA using high capacity cDNA reverse transcription kit (Applied Biosystems, Foster city, CA, USA). Real-time PCR was performed on 7500 Fast Real-Time PCR System (Applied Biosystems, Foster City, CA, USA) using TaqMan Gene Expression Master Mix (Applied Biosystems, Foster city). Each reaction contained 25 ng/µl cDNA that was amplified with Inventoried TaqMan Gene Expression Assay products (CERK: Assay ID: Hs00368483_m1; ITGAM (CD11b): Assay ID: Hs00355885_m1; ITGX (CD11c): Assay ID: Hs00174217_m1; (HLA-DR): Assay ID: Hs00219575_m1; (IL-1β): Assay ID: Hs01555410_m1; CCL2 (MCP-1): Assay ID: Hs00234140_m1; and GAPDH: Assay ID: Hs03929097_g1;). The threshold cycle (Ct) values were normalized to the house-keeping gene GAPDH, and the amounts of target mRNA relative to control were calculated with ΔΔCt-method^16–19^. Relative mRNA expression was expressed as fold expression over average of control gene expression. The expression level in control treatment was normalized to 1. Values are presented as mean ± SEM. Results were analyzed statistically; p < 0.05 was considered significant.

### Extracellular staining-flow cytometry

Monocytic cells were seeded in 24 well plate at 0.5 × 10^5^ cell/ml in serum free medium overnight. Cells were treated with CERK inhibitor NVP-231 (12 nM for 1 h or 0.01% DMSO (vehicle), then subjected to stimulation with TNF-α (10 ng/ml) or BSA (vehicle) for 6 h. Monocytic cells (1 × 10^6^ cells) were resuspended in FACS staining buffer (BD Biosciences) and blocked with human IgG (Sigma; 20 μg) for 30 min on ice. Cells were washed and resuspended in 100 μl of FACS buffer and incubated with CD11b (D12)-APC (cat# 340936; BD Biosciences), CD11b-FITC (cat# 6602573; Beckman Coulter), CD11c (S_HCL-3)-PE (cat# 347637; BD Biosciences), CD11c PE-Cy7 (cat # 117317; BD Biosciences) and CD14-APC (cat# 561708; BD Biosciences) on ice for 30 min. Cells were washed three times with FACS buffer and resuspended in 2% paraformaldehyde. Cells were centrifuged and resuspended in FACS buffer for FACS analysis (FACSCanto II; BD Bioscience, San Jose, USA). FACS data analysis was performed using BD FACSDiva Software 8 (BD Biosciences, San Jose, USA). The data for all flow cytometric measurements were expressed as staining index (SI) which is the ratio of the separation between the positive population (vehicle in blue or treatment in pink) and the negative population (non-stained cells in grey), divided by two times the standard deviation of the negative population (non-stained cells in grey). The mean staining index weas calculated from the three biological replicates minimum for each staining.

### Intracellular staining-flow cytometry

Flow cytometry analysis was used to investigate the expression of signaling pathway markers. Briefly, cells were seeded in 24 well plate at 0.5 × 10^5^ cells/ml in serum free medium overnight. Cells were treated with CERK inhibitor NVP-231 or DMSO (vehicle), then subjected to stimulation with TNF-α (10 ng/ml) or BSA (vehicle) for 10 min. After stimulation, cells were collected and washed. Cells were then incubated with fixation/permeabilization buffer (cat# 00-5523-00, eBioscience, San Diego, CA, USA) for 20 min on wet ice, followed by washing and staining with the following antibodies: mouse anti-human JNK-PE/ (pT183/pY185) (cat# 562480), mouse anti-human p38 MAPK/ (pT180/pY182) (cat# 612280), anti-human NF-κB P65-PE (cat # 558423; BD Biosciences) or Alexa Fluor 647 mouse anti-IκBα (cat # 560817; BD Biosciences) for 30 min. The cells were then washed and resuspended in PBS supplemented with 2% FCS for FACS analysis (FACSCanto II; BD Bioscience, San Jose, USA). FACS data analysis was performed using BD FACSDiva Software 8 (BD Biosciences, San Jose, USA). The data for all flow cytometric measurements were expressed as staining index (SI)^20^ which is the ratio of the separation between the positive population (vehicle in blue or treatment in pink) and the negative population (non-stained cells in grey), divided by two times the standard deviation of the negative population (non-stained cells in grey). The mean staining index was calculated from the three biological replicates minimum for each staining.

### IL-1β and MCP-1 determination

Secreted IL-1β and MCP-1 proteins were quantified in the supernatants of monocytic cells stimulated with TNF-α using sandwich ELISA, following the manufacturer’s instructions (R&D systems, Minneapolis, USA).

### Small interfering RNA (siRNA) transfections

Monocytes were washed and resuspended in 100 μl of nucleofector solution (Amaxa Noclecfector Kit V) and cells were transfected separately with siRNA-CERK (30 nM; OriGene Technologies, Inc. MD, USA), scrambled (control) siRNA (30 nM; OriGene Technologies, Inc. MD, USA, USA), and pmaxGFP (0.5 μg; Amaxa Noclecfector Kit V for THP-1, Lonza). All transfection experiments were performed with Amaxa Cell Line Nucleofector Kit V for monocytic cells (Lonza, Germany) by using Amaxa Electroporation System (Amaxa Inc, Germany), according to the manufacturer’s protocol^21^. After 36 h of transfection, cells were treated with TNF-α for 2 h. Cells were transfected with 20 nM of siRNA using Viromer Blue (Lipocalyx, Halle, Germany) as per the manufacture’s instruction. Cells were harvested for RNA isolation and staining studies. CERK gene knock down level was assessed by Real-Time PCR using CERK gene specific primer (CERK: Assay ID: Hs00368483; ThermoFisher Scientific).

### Measurement of NF-κB/AP-1 activity

We used NF-κB/AP-1 reporter monocytes (THP-1 XBlue; InvivoGen, San Diego, CA) stably transfected with a reporter construct expressing a secreted embryonic alkaline phosphatase (SEAP) gene under the control of a promoter inducible by transcription factors NF-κB and AP-1. Cells were stimulated with TNF-α (10 ng/ml) for 6–12 h at 37 °C. Levels of SEAP were detected in culture supernatants after 3 h incubation of supernatants with Quanti-Blue solution (InvivoGen, San Diego, CA, USA) and optical density (OD) was measured at 650 nm wavelength by ELISA reader.

### Western blotting

THP-1 cells were harvested and incubated for 30 min with lysis buffer containing Tris (62.5 mM, pH 7.5), 1% Triton X-100, and 10% glycerol. The lysates were centrifuged at 14,000 × *g* for 10 min and supernatants were collected. Protein concentration in lysates was measured by Quickstart Bradford Dye Reagent, 1× Protein Assay kit (Bio-Rad Laboratories, Inc, CA). Protein samples (20 µg each) were mixed with sample loading buffer, heated for 5 min at 95 °C and resolved by 12% SDS-PAGE. Cellular proteins were transferred to Immuno-Blot PVDF membrane (Bio-Rad Laboratories, USA) by electroblotting. The membranes were blocked with 5% non-fat milk in PBS for 1 h, followed by incubation with primary antibodies against p-SAPK/JNK, p-p38, p-NF-κB, SAPK/JNK, p38 and NF-κB in 1:1000 dilution at 4 °C overnight. All primary antibodies were purchased from Cell Signaling (Cell Signaling Technology, Inc). The blots were then washed four times with TBS and incubated for 2 h with HRP-conjugated secondary antibody (Promega, Madison, WI, USA). Immunoreactive bands were developed using an Amersham ECL plus Western Blotting Detection System (GE Health Care, Buckinghamshire, UK) and visualized by Molecular Imager ChemiDoc Imaging Systems (Bio-Rad Laboratories, Hercules, CA, USA)^22–24^.

### Statistical analysis

Statistical analysis was performed using GraphPad Prism software (La Jolla, CA, USA). Data are shown as mean ± standard error of mean, unless otherwise indicated. Unpaired Student t-test and one-way ANOVA followed by Tukey’s test were used to compare group means. For all analyses, data from a minimum of three replicates were used for statistical calculation. p-value < 0.05 was considered statistically significant. NS: Non-significant, *p < 0.05, **p < 0.01, ***p < 0.001 and ****p < 0.0001).

## Results

### CERK inhibition reduces the TNF-α-induced inflammatory responses in monocytes

**Figure 1:**
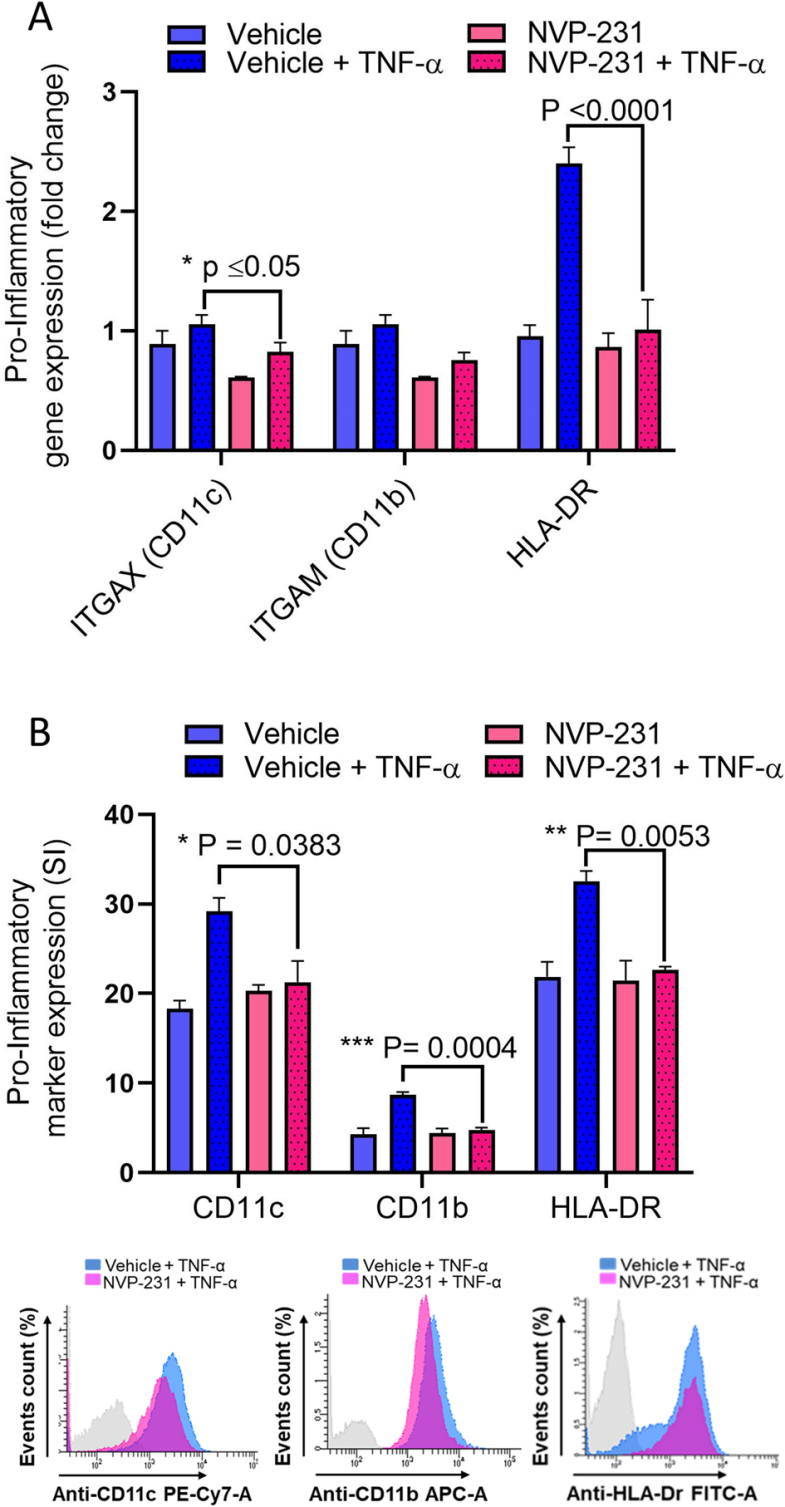
CERK inhibition blocks the TNF-α mediated pro-inflammatory changes in THP-1 cells. THP-1 cells were pretreated with CERK inhibitor (NVP-231: 12 nM) or vehicle for 1 h and then incubated with TNF-α for 2 h. Cells were harvested and mRNA expression of CD11c, CD11b and HLA-DR was determined by real-time RT-PCR (**A**). After 6 h treatment with TNF-α, cells were stained with antibodies against CD11c, CD11b ad HLA-DR along with isotype-matched control antibody. Surface expression was assessed by flow cytometry (**B**); data are presented as a bar graph of mean staining index, and representative histograms. All data are expressed as mean ± SEM (n ≥ 3). *p ≤ 0.05, **p ≤ 0.01, ***p ≤ 0.001, ****p ≤ 0.0001 versus vehicle.

CERK and its byproduct C1P are involved in many pathophysiological inflammatory processes^6^. To investigate whether CERK is involved in TNF-α-mediated immune responses, THP-1 monocytic cells were pre-treated with specific CERK inhibitor NVP-231(12 nM; Supplementary Fig. S1) before exposure to TNF-α. Treatment with NVP-231, followed by exposure to TNF-α, caused a significant reduction in the expression of phenotypic monocyte inflammatory markers including CD11c and HLA-DR at mRNA level and CD11c, CD11b and HLA-DR at protein level (Fig. 1A,B).

**Figure 2:**
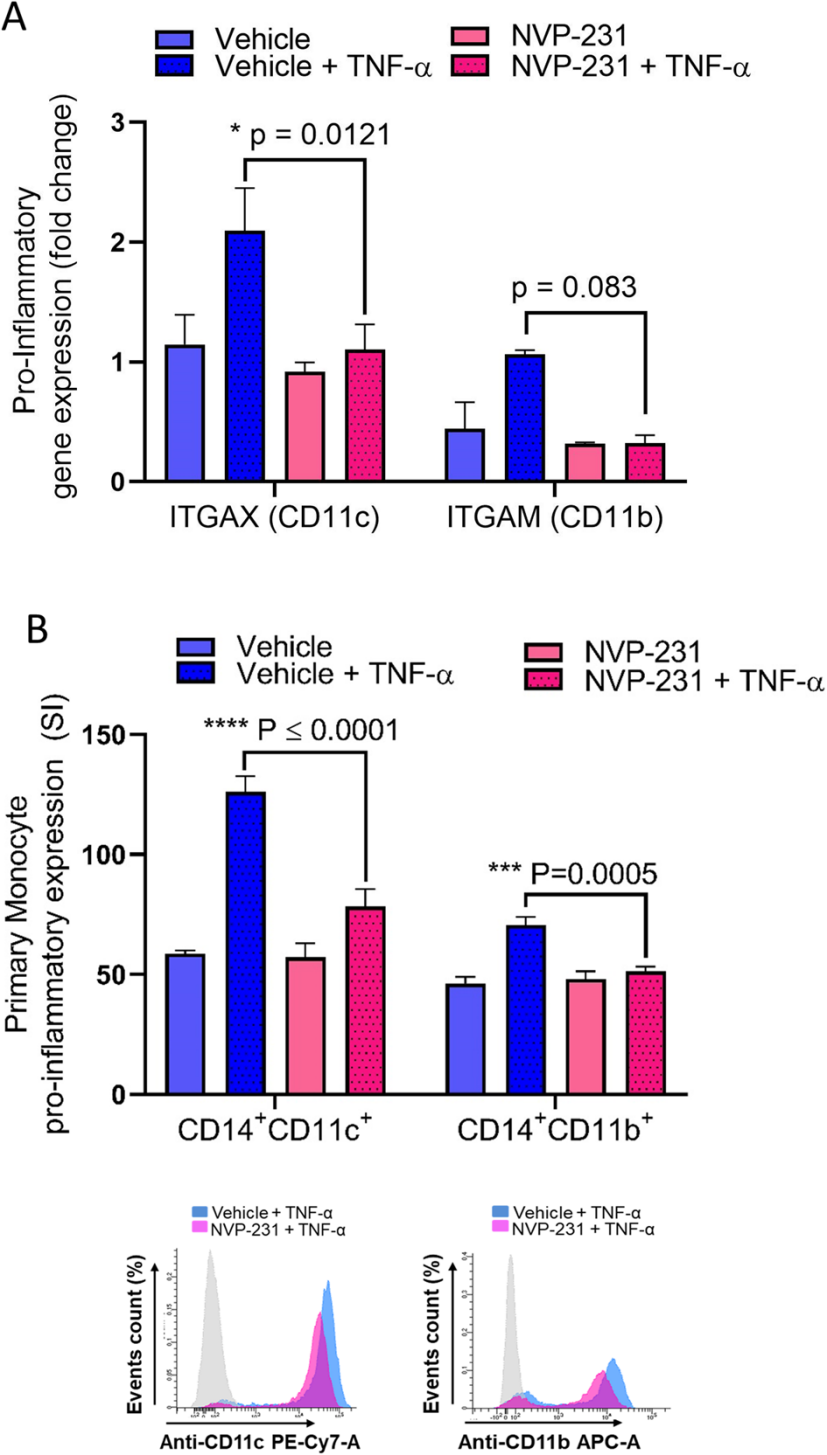
CERK inhibition blocks the TNF-α-mediated pro-inflammatory changes in primary human monocytes. Human primary monocytes were pretreated with CERK inhibitor (NVP-231: 12 nM) or vehicle for 1 h and then incubated with TNF-α for 2 h. Cells were harvested and mRNA expression of CD11c and CD11b was determined by real-time RT-PCR (**A**). After 6 h treatment with TNF-α, cells were stained with antibodies against CD11c, CD11b and CD14 along with isotype-matched control antibody. Surface expression of CD14^+^CD11c^+^ and CD14^+^CD11b^+^ was assessed by flow cytometry (**B**); data are presented as a bar graph of mean staining index, and representative histograms. All data are expressed as mean ± SEM (n ≥ 3). *p ≤ 0.05, **p ≤ 0.01, ***p ≤ 0.001, ****p ≤ 0.0001 versus vehicle.

To further validate the physiological relevance of these data obtained from THP-1 cells, primary human monocytes were preincubated with NVP-231, followed by exposure to TNF-α. Our results show that NVP-231 treatment inhibited the TNF-α-induced CD11c gene expression as well as CD14^+^CD11c^+^ and CD14^+^CD11b^+^ surface expression in primary human monocytes (Fig. 2A,B, respectively**)**. Our data also show that TNF-α slightly increases CERK expression at protein levels in monocytic cells (Supplementary Fig. S2). Collectively, these results suggest that CERK is involved in the TNF-α-mediated upregulation of pro-inflammatory response in human monocytic cells.

### CERK inhibition reduces the TNF-α-mediated IL-1β and MCP-1 production

**Figure 3:**
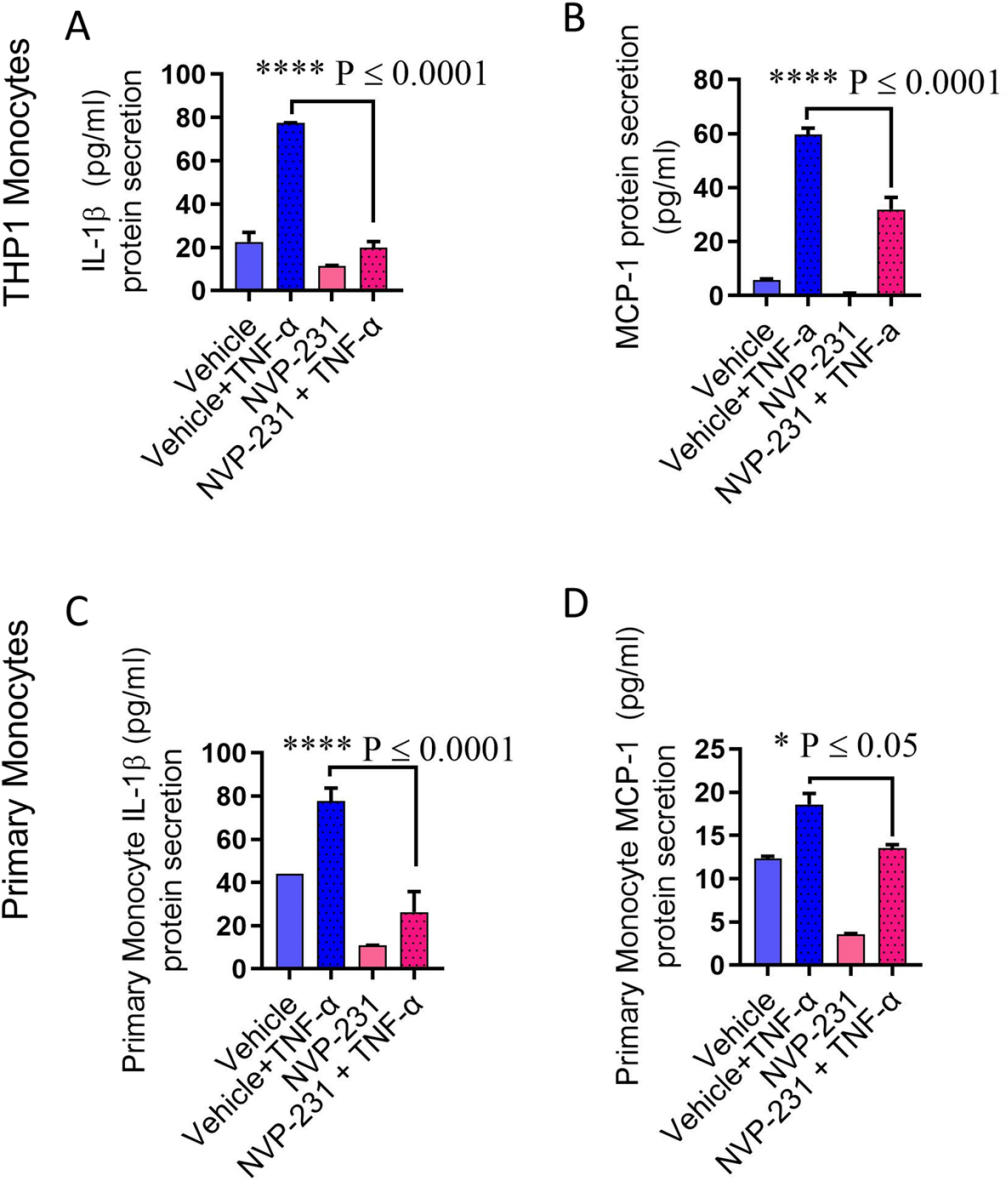
CERK inhibition suppresses the TNF-α-induced expression of IL-1β and MCP-1 in monocytes. Monocytic cells (primary monocytes, THP1 cells) were pretreated with CERK inhibitor (NVP-231: 12 nM) or vehicle for 1 h and then incubated with/without TNF-α for 12 h. Secreted IL-1β and MCP-1 proteins in culture media were determined by ELISA. IL-1β and MCP1 secreted by THP-1 cells (**A**,**B**), and primary monocytes (**C**,**D**). All data are expressed as mean ± SEM (n ≥ 3). *p ≤ 0.05, **p ≤ 0.01, ***p ≤ 0.001, ****p ≤ 0.0001 versus vehicle.

IL-1β and MCP-1 produced by activated monocytes contribute to the pathogenesis of different inflammatory conditions^25–27^. Next, we asked whether IL-1β and MCP-1 expression induced by TNF-α in THP-1cells or in primary monocytes, was reduced by the inhibition of CERK. Our data show that the inhibition of CERK in THP-1 monocytes significantly reduced the gene expression and production of IL-1β and MCP-1 following TNF-α stimulation (Fig. 3A,B, Supplementary Fig. S3A,B). As expected, similar results were observed with primary monocytes (Fig. 3C,D). These results indicate that TNF-α regulates the production of key inflammatory cytokines, such as IL-1β and MCP-1, in monocytes through the activation of CERK.

### CERK downregulation suppresses the TNF-α-mediated pro-inflammatory responses in human monocytes

**Figure 4:**
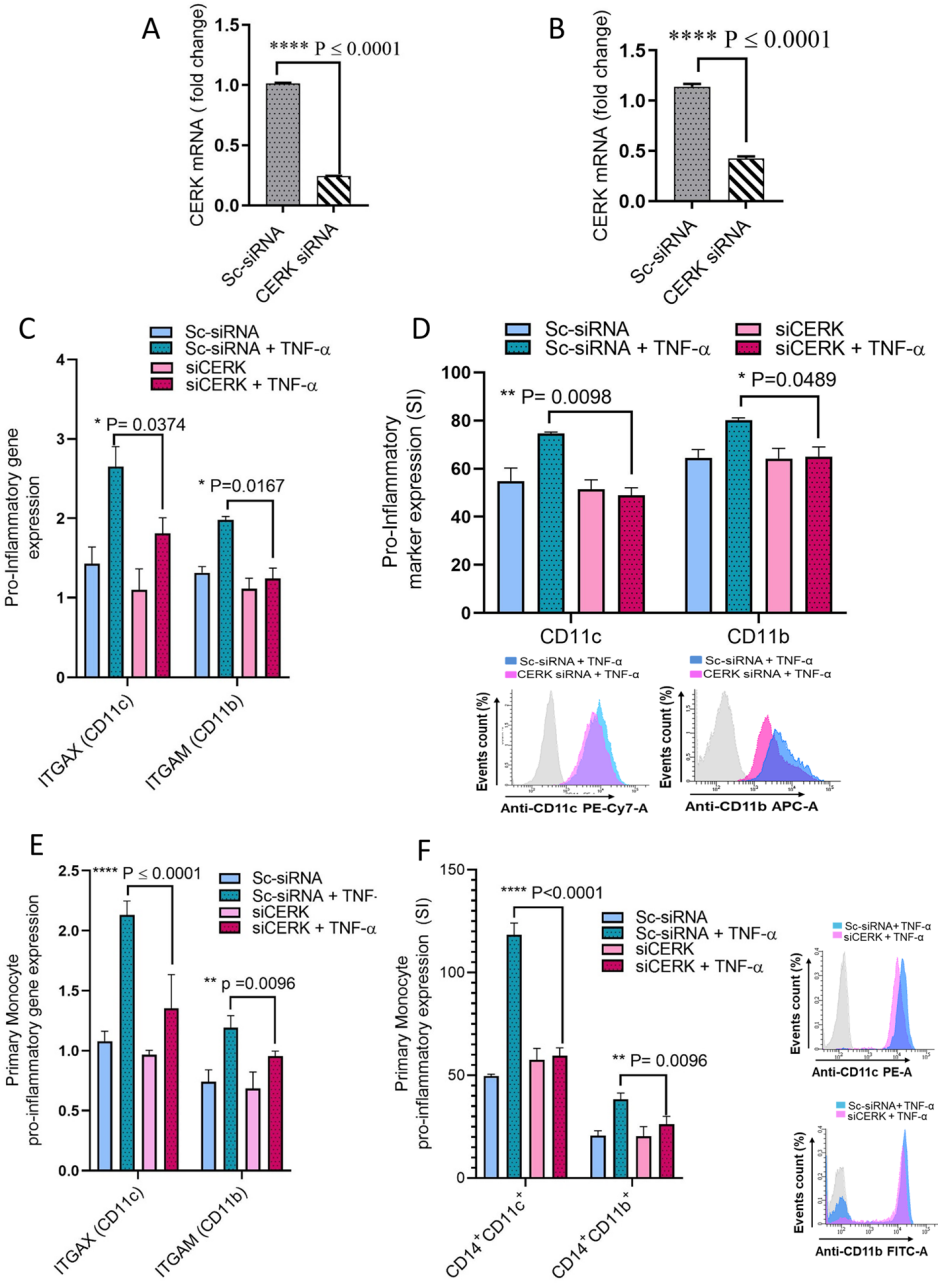
TNF-α mediated pro-inflammatory monocytic responses require CERK. THP-1 monocytes and primary human monocytes were transfected with scrambled-siRNA (negative control; NC) or CERK siRNA and incubated for 36 h. Real-time RT-PCR was performed to measure (**A**) CERK mRNA and protein expression in THP-1 monocytic cells and (**B**) primary monocytes. CERK-downregulated THP-1 cells were treated with TNF-α and vehicle. Cells were stained with antibodies against CD11c and CD11b along with isotype-matched control antibody and surface expression of these proteins was measured by flow cytometry. Flow cytometry data are presented as a bar graph of mean staining index of the selected inflammatory markers (**C**). CD11c and CD11b were determined by real-time RT-PCR (**D**). Surface expression of CD14^+^CD11c^+^ and CD14^+^CD11b^+^ was determined in primary monocytes by flow cytometry (**E**) CD11c and CD11b mRNA expression was determined by real-time RT-PCR (**F**). All data are expressed as mean ± SEM (n ≥ 3). *p ≤ 0.05, **p ≤ 0.01, ***p ≤ 0.001, ****p ≤ 0.0001 versus vehicle.

**Figure 5:**
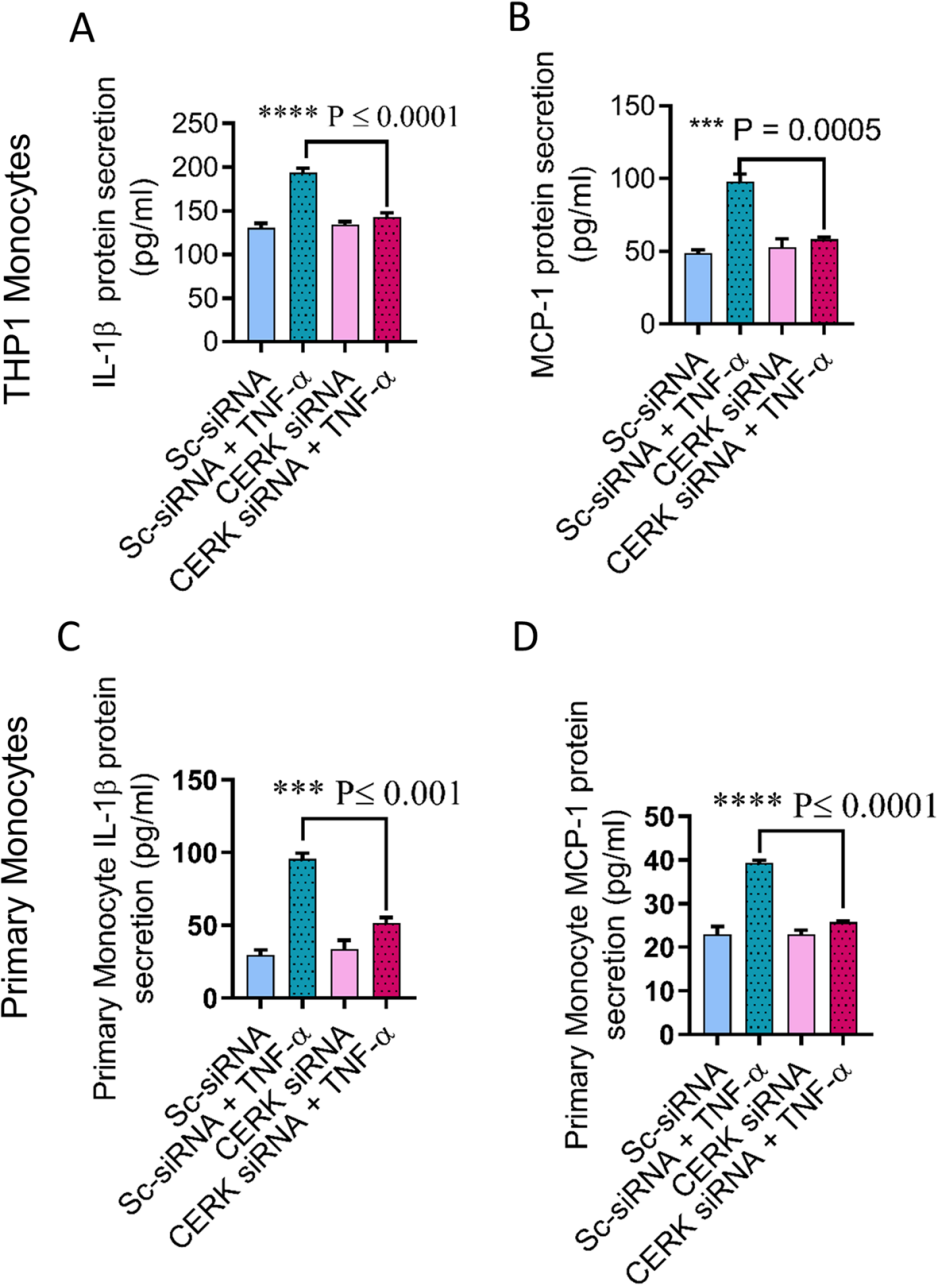
CERK downregulation attenuates the IL-1β and MCP-1 secretion in TNF-α activated monocytes. THP-1 monocytes and primary human monocytes were transfected with scrambled-siRNA (negative control; NC) or CERK siRNA and incubated for 36 h. CERK-downregulated THP-1 cells and primary human monocytes were treated with TNF-α for 12 h. Secreted IL-1β and MCP-1 proteins were measured in culture media using ELISA. IL-1β and MCP-1 secreted by THP-1 cells (**A**,**B**), and primary human monocytes (**C**,**D**). All data are expressed as mean ± SEM (n ≥ 3). *p ≤ 0.05, **p ≤ 0.01, ***p ≤ 0.001, ****p ≤ 0.0001 versus vehicle.

To further confirm the role of CERK in TNF-α-induced inflammatory alterations in THP-1 monocytic cells and primary human monocytes, cells were transfected with siRNA against CERK, reducing CERK mRNA levels by 50–70% as compared with scrambled (control) siRNA (Fig. 4A,B, Supplementary Fig. S4). The expression of CD11c and CD11b at both mRNA and protein levels, was significantly reduced in CERK-downregulated THP-1 monocytes (Fig. 4C,D) as well as primary monocytes (Fig. 4E,F) after exposure to TNF-α. Next, we wanted to see whether CERK downregulation in monocytic cells disrupted the TNF-α-induced secretion of IL-1β and MCP-1. As expected, CERK-downregulated monocytic cells failed to respond to TNF-α stimulation for production of IL-1β and MCP-1when compared to cells transfected with scrambled siRNA (Fig. 5A–D). Altogether, our results suggest the significant involvement of CERK in inflammatory responses induced by TNF-α in monocytic cells.

### TNF-induces ceramide 1 phosphate in monocytic cells

**Figure 6:**
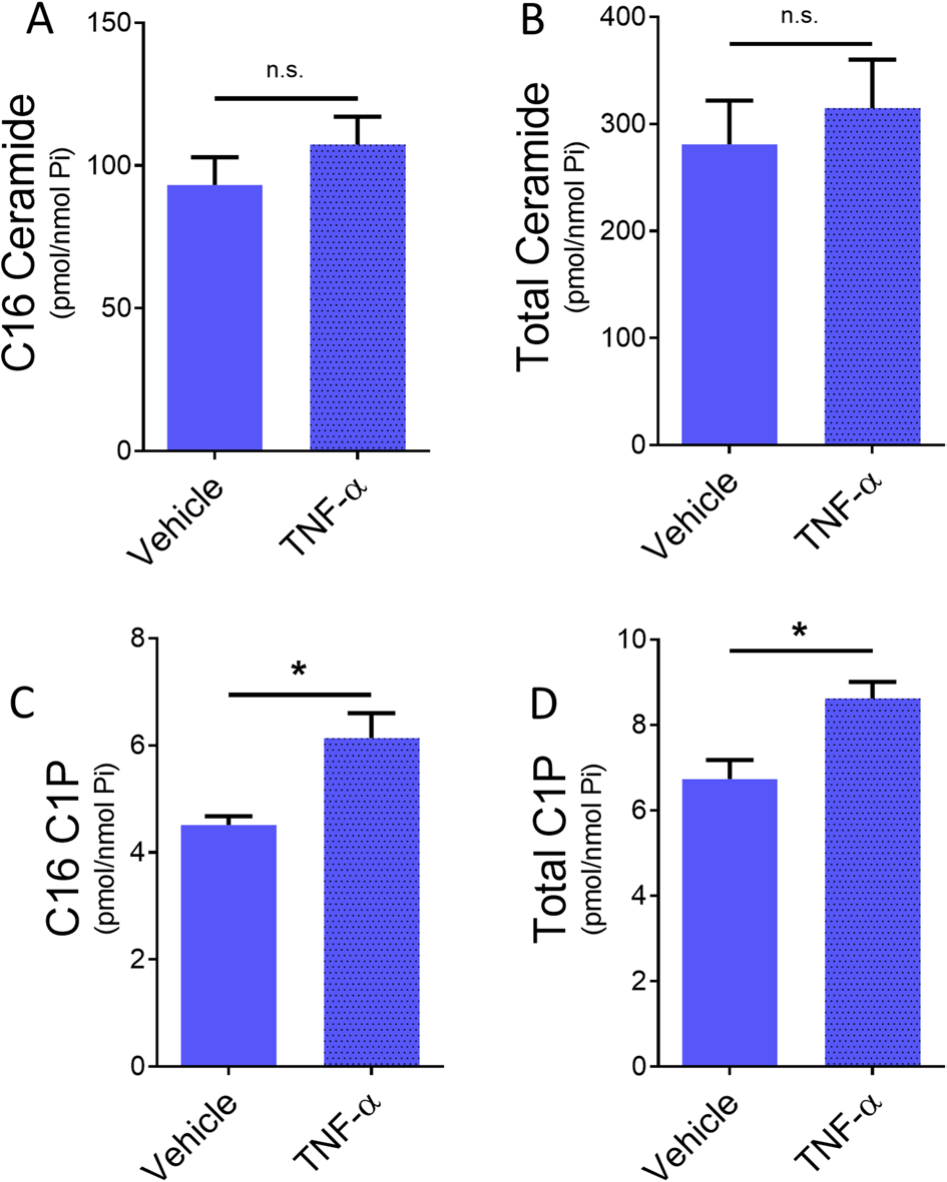
TNF-α induces ceramides in monocytic cells. THP-1 monocytes were treated with vehicle or TNF-α for 12 h. Cellular lipid levels were analyzed. (**A**) C16 ceramide, (**B**) total ceramides, (**C**) C16 C1P, and (**D**) total C1P by ESI/MS/MS at Stony Brook University Lipidomics Shared Resource Core and data were normalized to total lipid phosphate (Pi). Data represent mean ± SEM, n = 4, *p < 0.05 as compared to vehicle treatment.

Since our data show that CERK is involved in TNF-α-mediated monocytic cell activation and inflammatory responses, next we examined whether TNF-α induced the production of C1P. We measured the ceramides and C1P levels following TNF-α stimulation. While, TNF-α stimulation did not significantly alter C16 or total ceramide levels (Fig. 6A,B), C16-C1P and total C1P levels were significantly increased (Fig. 6C,D). These data suggest that TNF-α regulates the production of C1P.

### NVP-231 inhibits the TNF-α-induced activation of MAPKs and NF-κB

**Figure 7:**
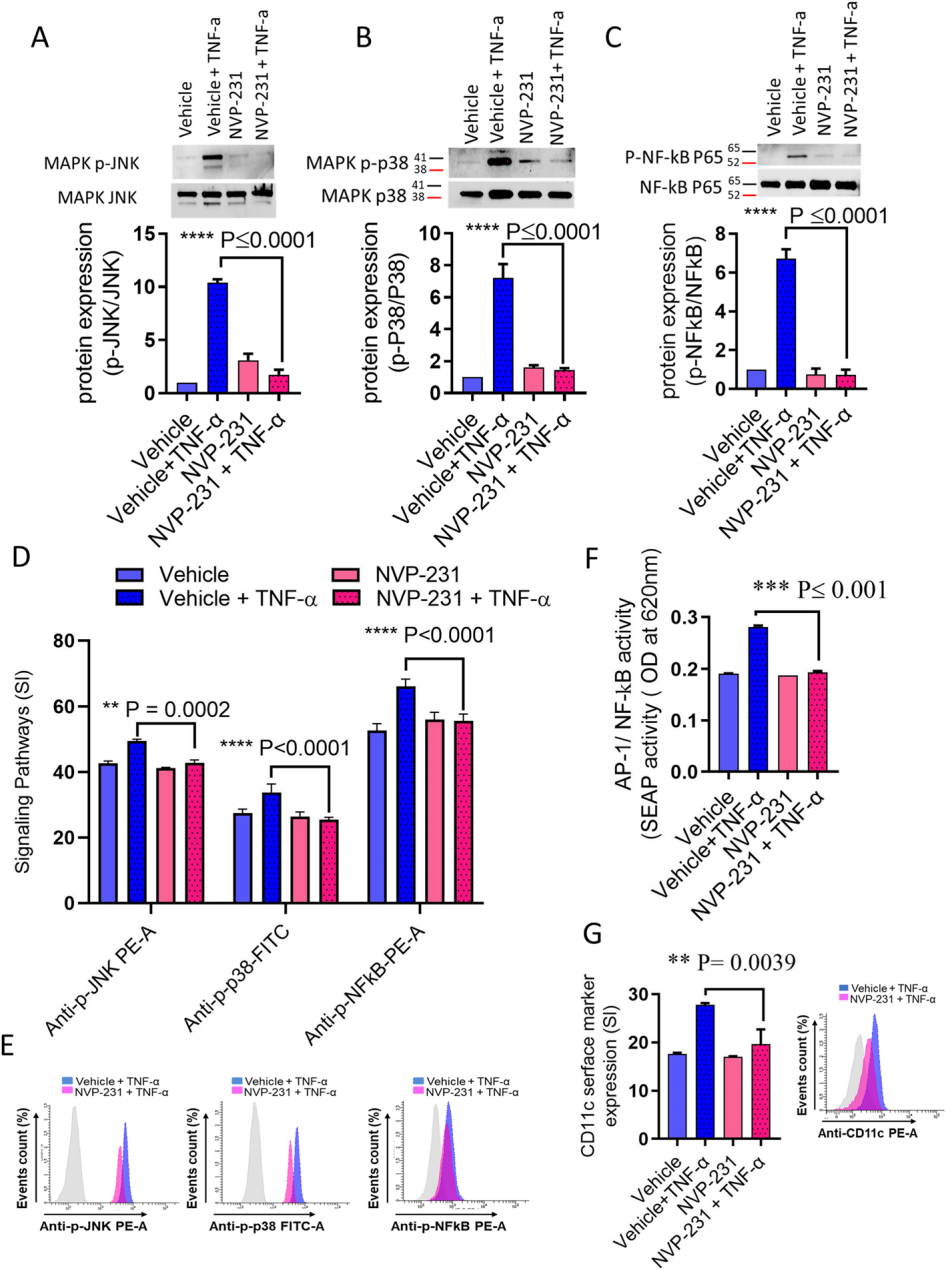
CERK inhibition downmodulates TNF-α-induced activation of MAPK and NF-κB signaling pathways in THP-1 cells. THP-1 monocytic cells were pretreated with CERK inhibitor (NVP-231: 12 nM) and then incubated with TNF-α. Cell lysates were prepared as described in “Materials and methods” section. Samples were run on denaturing gels. Immuno-reactive bands were developed using an Amersham ECL Plus Western Blotting Detection System (GE Healthcare, Chicago, IL, USA) and visualized by Molecular Imager ChemiDoc MP Imaging Systems (Bio-Rad Laboratories, Hercules, CA, USA). (**A**) Phosphorylated proteins of SPAK/JNK, (**B**) p38 and (**C**) NF-κB are shown in the upper panels with the lower panel representing respective total proteins. The phosphorylation intensity was quantified by using Image Lab software (version 6.0.1, Bio-Rad, Hercules, CA, USA) and presented in bar graphs as arbitrary unit (AU) of corrected protein expression. Signaling proteins were also determined by flow cytometry. Cell were immediately fixed and permeabilize for 20 min at 4 °C, then stained to visualize the JNK, p38 and NF-κB phosphorylation. Flow cytometry data are presented as a bar graph of mean staining index (SI) as well as by representative histograms (**D**,**E**). Bar graphs depict the mean values ± SEM of staining intensity (SI). P < 0.05 was considered as statistically significant (*p ≤ 0.05; **p ≤ 0.01, ***p ≤ 0.001, ****p ≤ 0.0001). The data in all figures are representative of three independent experiments. NF-κB/AP-1 reporter monocytic cells were pretreated with CERK inhibitor (NVP-231: 12 nM) or vehicle for 1 h and then incubated with TNF-α for 12 h. Cell culture media were assayed for SEAP reporter activity, representing NF-κB/AP-1 activation (**F**). Reporter cells were also tested for surface expression of CD11c (**G**).

Since CERK/C1P activate phosphorylation of JNK, p38 and NF-κB^28^, we questioned whether CERK was involved in TNF-α-induced activation of JNK, p38 and NF-κB signaling pathways. To investigate the CERK function in the TNF-α-mediated activation of MAPK and NF-κB signaling pathways, cells were pre-treated with CERK inhibitor NVP-231 prior to TNF-α stimulation. Our results show that the inhibition of CERK significantly decreased the TNF-α-mediated phosphorylation of JNK, p38, and NF-κB (Fig. 7A–E, Supplementary Fig. S5A–C). NF-κB and AP-1 are the downstream transcription factors of TNF-α signaling pathways. To further examine the role of CERK in TNF-α-mediated activation of NF-κB/AP-1, we used NF-κB/AP-1 activity reporter human monocytic cells. Our data show that TNF-α induces higher NF-κB/AP-1 activity in the reporter cells and this activity was diminished in the cells that were treated with CERK inhibitor (Fig. 7F). Concordantly, TNF-α-stimulated reporter cells also showed elevated surface expression of the activation marker CD11c which was also suppressed when the cells were treated with CERK inhibitor (Fig. 7G). Together, these data support the role of CERK in TNF-α-mediated activation of NF-κB and AP-1 transcription factors.

### Increased gene expression of CERK and TNF-α in PBMCs of obese individuals

**Figure 8:**
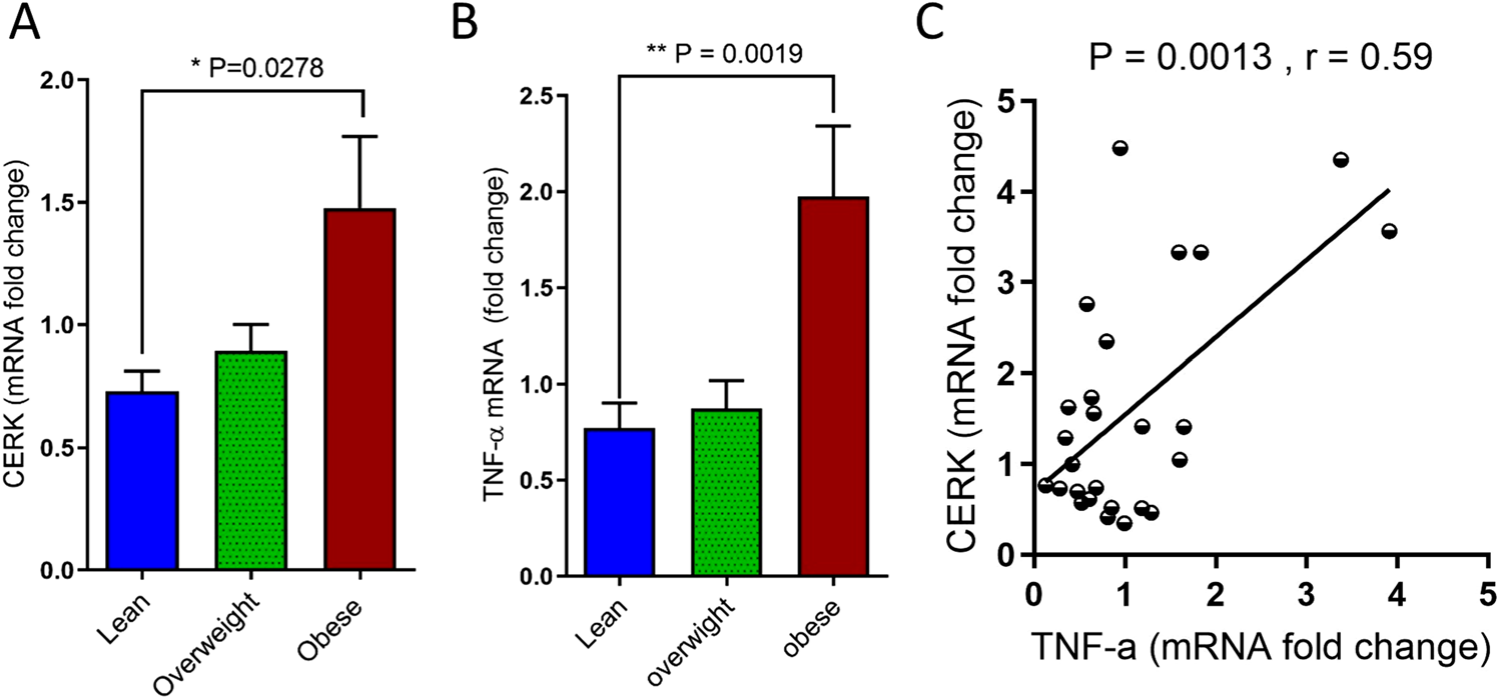
Association between TNF-α and CERK expression in PBMCs from obese individuals. PBMCs were isolated from human peripheral blood samples obtained from lean (n = 13), overweight (n = 14) and obese (n = 13) individuals. CERK and TNF-α mRNA expression was detected by real-time RT-PCR and represented as fold change over controls (**A**,**B**). Pearson’s correlation coefficient (r) is shown between CERK and TNF-α (**C**).

Our in vitro data show the involvement of CERK in TNF-α-mediated inflammatory responses in human monocytic cells and primary human macrophages. Next, we asked if these data were relevant to a clinical setting, depicting the CERK-TNF-α modulations in a metabolic disorder. Therefore, we determined the expression levels of CERK and TNF-α in PBMCs of lean, overweight, and obese individuals. To this end, we isolated total RNA from PBMCs of 26 individuals including lean, overweight, and obese, and determined the gene expression CERK and TNF-α. Our data show that expression of both CERK and TNF-α was elevated in obese as compared to lean individuals (Fig. 8A,B, respectively**).** Furthermore, CERK expression positively correlated with that of TNF-α (r = 0.59; p = 0.0013 (Fig. 8C)).

## Discussion

In the present study, we report that CERK plays a role in the regulation of TNF-α-induced inflammatory responses in monocytic cells. Our data show that pharmacologic inhibition of CERK with NVP-231^29^ or genetic silencing with CERK-specific siRNA significantly suppressed the expression of inflammatory markers, such as CD11b, CD11c and HLA-DR on human monocytes stimulated with TNF-α. TNF-α-mediated inflammatory immune cell polarization contributes to the pathogenesis of many inflammatory diseases^30–32^. Elevated levels of TNF-α are persistently expressed in obesity and metabolic syndrome. Of note, obese mice and humans show increased numbers of monocytes/macrophages with the elevated expression of CD11c marker as well as MCP-1 production under the influence of diet-induced obesity. On the other hand, CD11c deficiency in mice led to decreased inflammation in the animals, marked by reduced expression of inflammatory markers including HLA-DR, CCL5, and CCL4^33^. CD11b expression was reported to be elevated in obese individuals which associated with the development of metabolic syndrome^34^. CD11c expression was found to be higher in adipose-resident HLA-DR^+^ macrophages from obese women^35^. Since TNF-α is invariably elevated in obesity and metabolic syndrome, expression of inflammatory phenotypic markers in monocytes/macrophages could have been induced by TNF-α.

Given that CERK has been suggested to have roles in inflammation, though not clearly defined, we set out to determine the possible involvement of CERK in TNF-α-induced inflammation. The results showing TNF-α-induced C1P levels in monocytic cells suggest activation of CERK. Interestingly, we previously found that TNF-α induced nSMase2 activity, leading to elevated expression of proinflammatory response markers including CD11c, IL-1β, and MCP-1 in monocytes/macrophages. We further showed that nSMase2 inhibitor GW4869 blocked the phosphorylation of ERK1/2, JNK, c-Jun and NF-κB together with suppression of NF-κB/AP-1 activity^36^. Thus, our previous study deciphers TNF-α effect on proinflammatory responses via the nSMase2-ceramide axis, while the present study demonstrates TNF-α effects on proinflammatory responses (NF-κB, p38, and JNK phosphorylation as well as NF-κB/AP-1 activity) in monocytes via the CERK-C1P axis. Inflammatory role of C1P was first reported in lung adenocarcinoma cells; C1P stimulated the release of arachidonic acid in lung cancer cells and led to the production of eicosanoids which are implicated in inflammation^37^. C1P-mediated inflammation is directly regulated by activation of cytosolic phospholipase-A2a (cPLA2a), an enzyme that releases AA from membrane phospholipids^38^. CERK KO mice expressed decreased levels of proinflammatory cytokines as well as less macrophage infiltration in adipose tissue when fed high fat diets compared to their wild-type counterparts^39^. Our findings specifically show that CERK inhibition or its downregulation in monocytic cells significantly blocks the production of critical inflammatory mediators including IL-1β and MCP-1. We reported previously in one of our study that knockdown of CERK led to a significant decrease in CCL5 mRNA and protein expression following TNF-α stimulation^40^. IL-1β and MCP-1 are the major proinflammatory mediators produced mostly by the activated monocytes/macrophages. Accumulating evidence confirms that IL-1β and MCP-1 are critically involved in obesity-associated inflammation in the rodent models^41,42^. IL-1β and MCP-1 are expressed in the human adipose tissues, but primarily by the nonfat cells^41^. In obese mice and humans, adipose tissue IL-1β and MCP-1 mRNA expression as well as their circulating levels correlated positively with insulin resistance. On the other hand, IL-1β and MCP-1 inhibition reduced the insulin resistance and adipose tissue inflammation in obese mice^43^. Furthermore, IL-1β and MCP-1 produced by TNF-α-stimulated mouse adipocytes/monocytes were shown to induce insulin resistance in the liver and adipose tissues^44,45^.

MAPKs and NF-κB signaling pathways get potently activated in the cells treated with TNF-α. These pathways are commonly involved in the regulation of various inflammatory mediators that associate with the pathogenesis of different inflammatory diseases^46^. Importantly, MAPK signaling molecules cooperate with each other as well as with other inflammatory pathways including NF-κB to elicit immune responses that are implicated in metabolic inflammation^47,48^. We previously reported the role of ERK, JNK and NF-κB signaling in the regulation of TNF-α-mediated expression of multiple inflammatory mediators such as IL-8, MCP-1 and MIP-1α in monocytes^36,49,50^. These studies collectively point to the significance of MAPK and NF-κB pathways in inflammation through the regulation of various inflammatory mediators. Similarly, TNF-α-induced expression of CD11c and IL-1β in the lungs of mice involved the p38 MAPK signaling^51^. It was shown that p38 signaling was implicated in nSMase2 phosphorylation and activation^52^. Our data are corroborated by a previous study^40^ showing that disrupting CERK activity by either NVP-231 or siRNA decreases the TNF-α-induced phosphorylation of p38 MAPK, JNK, and NF-κB. TNF-α binding to its cognate receptor on cell surface phosphorylates the downstream c-Jun and NF-κβ, resulting in the activation of several inflammatory genes. In line with these observations, we show that CERK inhibition by NVP-231 significantly suppresses the TNF-α-induced NF-κB/AP-1 activity in the reporter monocytic cells, pointing to a critical role of CERK in TNF-α-induced inflammatory responses in monocytic cells. It may be further noted that although TNF-α-dependent activation of NF-κB has been well studied, the CERK-dependent phosphorylation of NF-κB and other transcription factors (p38 MAPK and JNK) represents another proinflammatory cascade which is activated by TNF-α stimulation of monocytic cells. In agreement with our data, a study reported that C1P regulated cell survival via activating the PI3K/PKB/NF-κB signaling pathway in mammalian cells^53^. In addition, ERK phosphorylation was demonstrated in human osteoblastic cells that were stimulated with short-chain C1P^54^.

**Figure 9:**
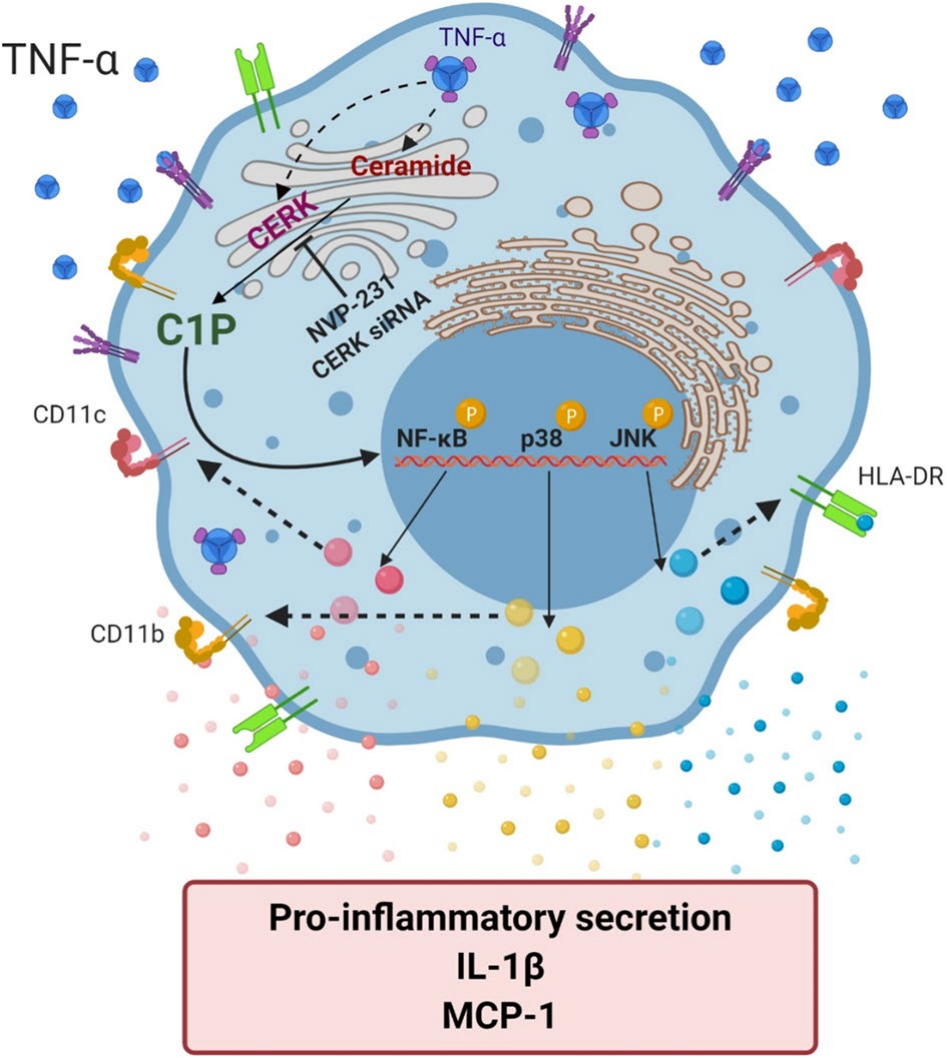
Schematic illustrating the involvement of CERK in TNF-α-mediated inflammatory responses in human monocytic cells. The figure was created using BioRender.com.

Overall, our data point to an interesting role of CERK and C1P in the regulation of TNF-α-induced inflammatory responses in monocytic cells through the mechanism involving MAPK and NF-κB signaling (Fig. 9). Given that these data show the reduced monocyte inflammatory responses following CERK inhibition or silencing, further studies will be required to validate whether CERK targeting can be useful to alleviate chronic inflammation and insulin resistance in metabolic disorders.

In conclusion, our study supports a novel role of CERK in the regulation of TNF-α-induced inflammatory responses in monocytic cells and primary human macrophages, involving the MAPK and NF-κB signaling pathways. We further point to the possible link between TNF-α and CERK in obesity that could promote metabolic inflammation.

## Supplementary Information

Supplementary Information.
