# Supplementary material for: Ceramide kinase regulates TNF-α-induced immune responses in human monocytic cells
Source: Sci Rep. 2021 Apr 15;11:8259. doi: 10.1038/s41598-021-87795-7 (PMC8050074; doi:10.1038/s41598-021-87795-7)

Title: **Ceramide Kinase Regulates TNF-α-Induced Immune Responses in Human Monocytic Cells**

Authors: Fatema Al-Rashed[1], Zunair Ahmad[2], Ashley J. Snider[3,4,] Reeby Thomas[1], Shihab Kochumon[1], Motasem Melhem[5], Sardar Sindhu[6], Lina M. Obeid[3], Fahd Al-Mulla[5], Yusuf A. Hannun[3], Rasheed Ahmad[1*]

Affiliations: [1]Immunology & Microbiology Department, Dasman Diabetes Institute, Kuwait; [2] Royal College of Surgeons in Ireland –Bahrain; [3]Stony Brook Cancer Center and Department of Medicine, Stony Brook University, Stony Brook, NY 11794, USA; [4]Department of Nutritional Sciences, College of Agriculture and Life Sciences, University of Arizona, Tucson, AZ 85721, USA; [5]Genetics and Bioinformatics Department, Dasman Diabetes Institute, Kuwait; [6]Animal and Imaging Core Facility, Dasman Diabetes Institute, Kuwait.

***Correspondence:** Rasheed Ahmad Ph.D., Immunology & Immunology Department, Dasman Diabetes Institute, Al-Soor Street, Kuwait, P.O. Box 1180 Dasman, 15462 Kuwait. Phone: 965 2224 2999 Ext. 4311; Fax: 965 2249 2406; E-mail: rasheed.ahmad@dasmaninstitute.org

Supplementary Figure 1

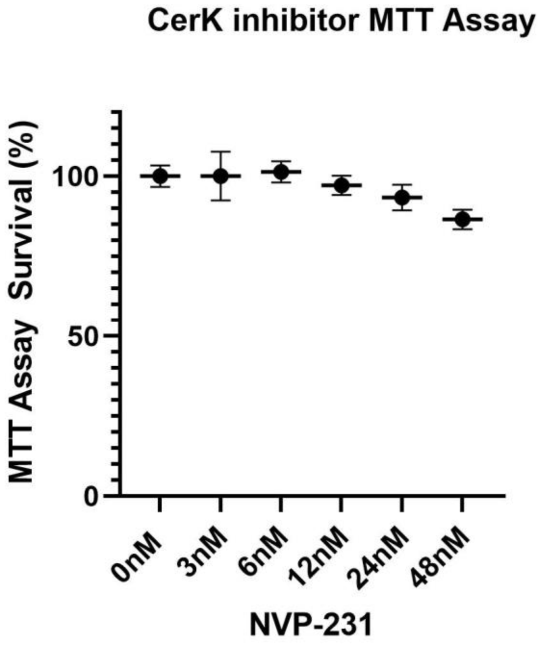

Figure S1. Cell viability assay

The cell viability was detected by MTT assay (TACS#4890-25-01, R&D systems, USA) as per manufacturer instructions. Briefly, THP-1 cells were plated in a 96-well plate at a density of 2×10^5 cells/ml, with 100 μl per well. Cells were treated with different concentrations of CERK inhibitor NVP-231 for 1h. Then 10 μl of MTT solution was added into each well. Cells were incubated at 37 °C for 4 h. 100 ul of solubilization solution (10% SDS in 0.01M HCL) was added to dissolve the formazan crystals. The optical density was measured at 570 nm and blanked at 650 nm (Synergy H4, BioTek, USA). Cell viability was calculated as the percentage of surviving fraction after exposure to NVP-231 relative to the mock control. All data are expressed as mean ± SEM (n=6).

Supplementary Figure.2

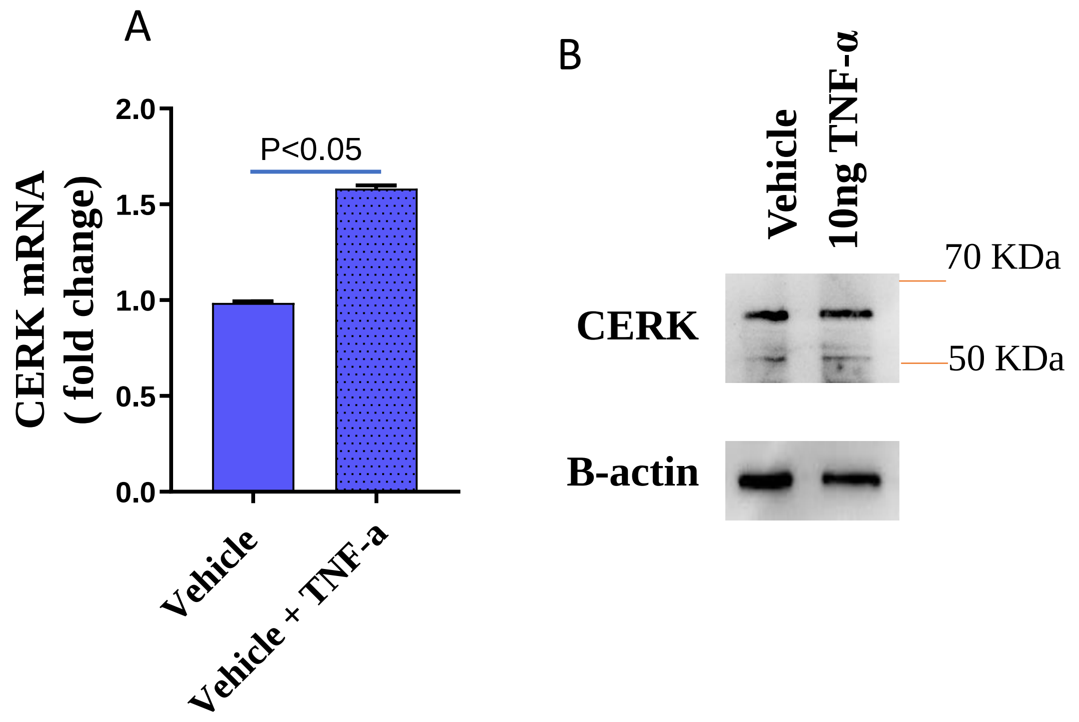

Figure S2 . Impact of TNF-α on CERK expression at both gene and protein levels in monocytic cells. Monocytic cells were treated with vehicle or TNF-α vehicle for 4 hours. **(A)** Gene expression of CERK was determined by real time RT-PCR. (**B**) For protein expression, cells were treated with vehicle or TNF-α for 12h. Cell lysates were prepared as described in material and methods. Samples were run on denaturing gels. The membranes were then blocked with 5% non-fat milk in PBS for 1h, followed by incubation with primary rabbit polyclonal antibody against CERK (abcam, ab 155061) in 1: 500 dilution at 4°C overnight. The blots were then washed four times with TBS and incubated for 2h with HRP-conjugated secondary antibody (Promega, Madison, WI, USA). Immuno-reactive bands were developed using an Amersham ECL Plus Westren Blotting Detection System (GE Healthcare, Chicago, IL, USA) and visualized by Molecular Imager ChemiDoc MP Imaging Systems (Bio-Rad Laboratories, Hercules, CA, USA). CERK is shown in the upper panel with the lower panel representing house keeping gene Beta-actin.
C)

Supplementary Figure 3

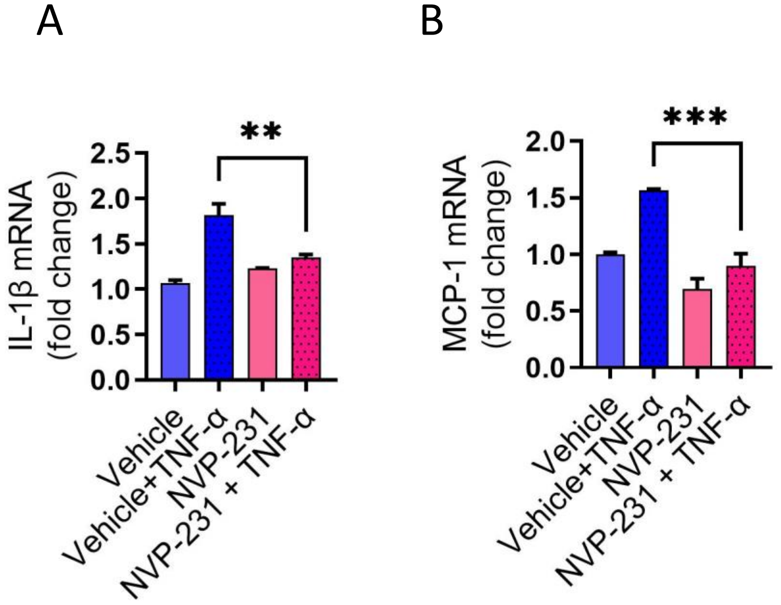

Figure S3. IL-1β and MCP-1 gene expression by TNF-α activated monocytes are suppressed by CERK inhibition.

Monocytic cells were pretreated with CERK inhibitor (NVP-231: 12nM) or vehicle for 1h and then incubated with TNF-α for 4h. Cells were harvested for total RNA isolation. Gene expression of IL-1β **(A**) and MCP-1 (**B**) was determined by real time RT-PCR. All data are expressed as mean ± SEM (n ≥ 3). **p≤0.01, ***p≤0.001 versus vehicle + TNF-α.

Supplemental Figure 4

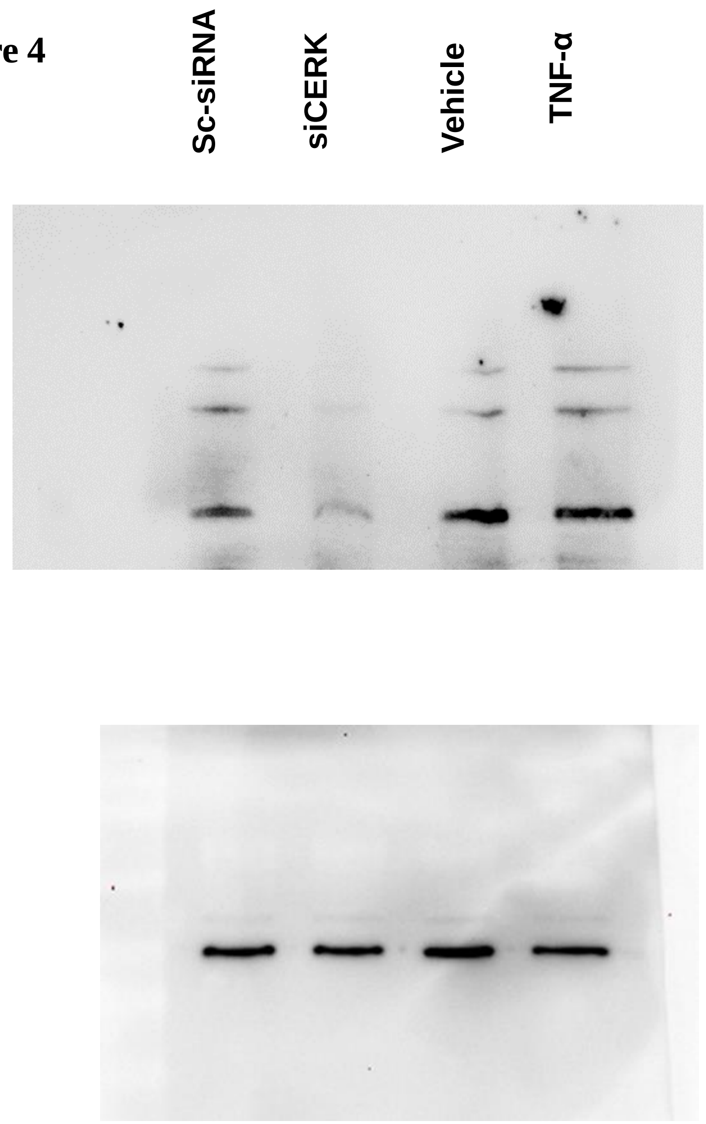

Figure S4. SiRNA transfection efficacy. THP-1 monocytic cells were transfected with scrambled-siRNA (negative control; NC) or CERK siRNA as described in material and methods. Cell lysates were prepared as described in material and methods. Samples were run on denaturing gels. The membranes were then blocked with 5% non-fat milk in PBS for 1h, followed by incubation with primary rabbit polyclonal antibody against CERK (abcam, ab 155061) at 1: 500 dilution or B-actin at 1: 1000 dilution (Cell Signaling Technology, Inc) at 4°C overnight. The blots were then washed four times with TBS and incubated for 2h with HRP-conjugated secondary antibody (Promega, Madison, WI, USA). Immuno-reactive bands were developed using an Amersham ECL Plus Westren Blotting Detection System (GE Healthcare, Chicago, IL, USA) and visualized by Molecular Imager ChemiDoc MP Imaging Systems (Bio-Rad Laboratories, Hercules, CA, USA). CERK is shown in the upper panel with the lower panel representing house keeping gene Beta-actin.

Supplemental Figure 5A-C (full western blot images for Figure 7):

THP1 monocytic cells were pretreated with CERK inhibitor (NVP-231: 12nM) and then incubated with TNF-α. Cell lysates were prepared as described in material and methods. Samples were run on denaturing gels. Immuno-reactive bands were developed using an Amersham ECL Plus Westren Blotting Detection System (GE Healthcare, Chicago, IL, USA) and visualized by Molecular Imager® ChemiDoc™ MP Imaging Systems (Bio-Rad Laboratories, Hercules, CA, USA). **(A)** Phosphorylated proteins of SPAK/JNK, **(B)** p38 and **(C)** NF-kB are shown in the upper panels with the lower panel representing respective total proteins.

Supplemental figure 5A

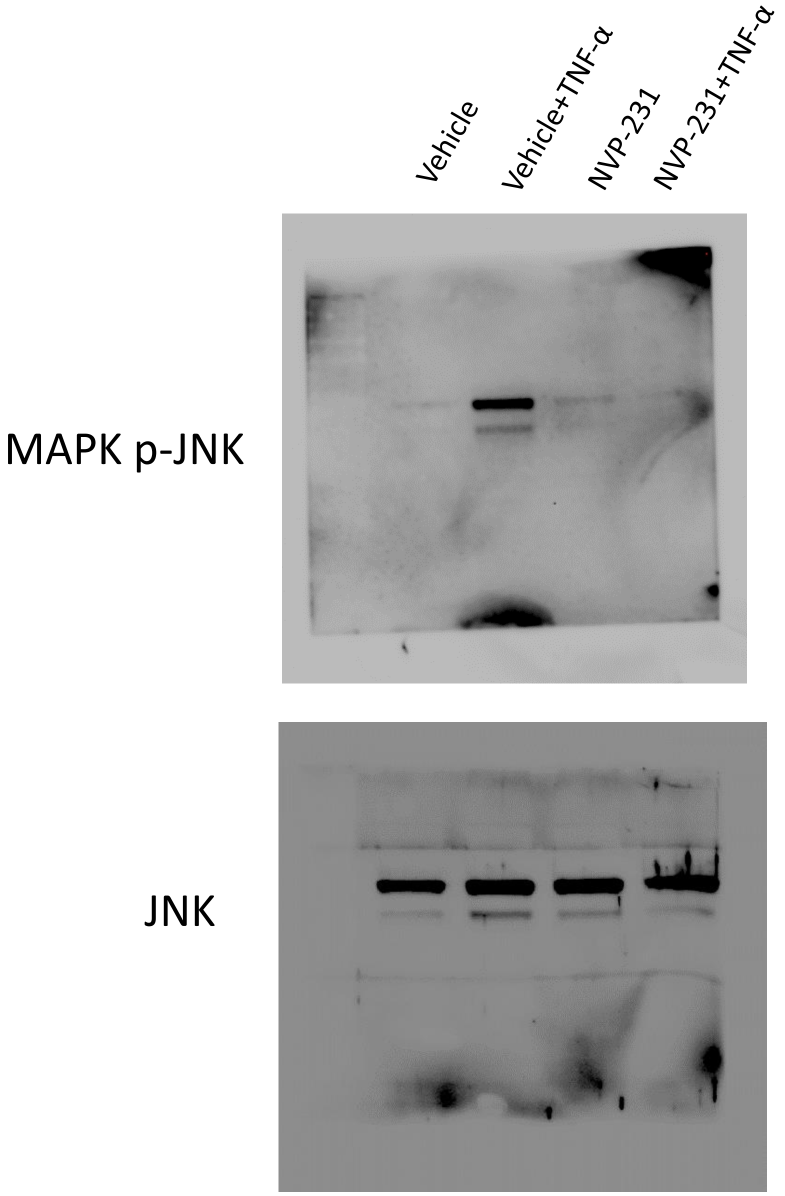

Supplemental figure 5B

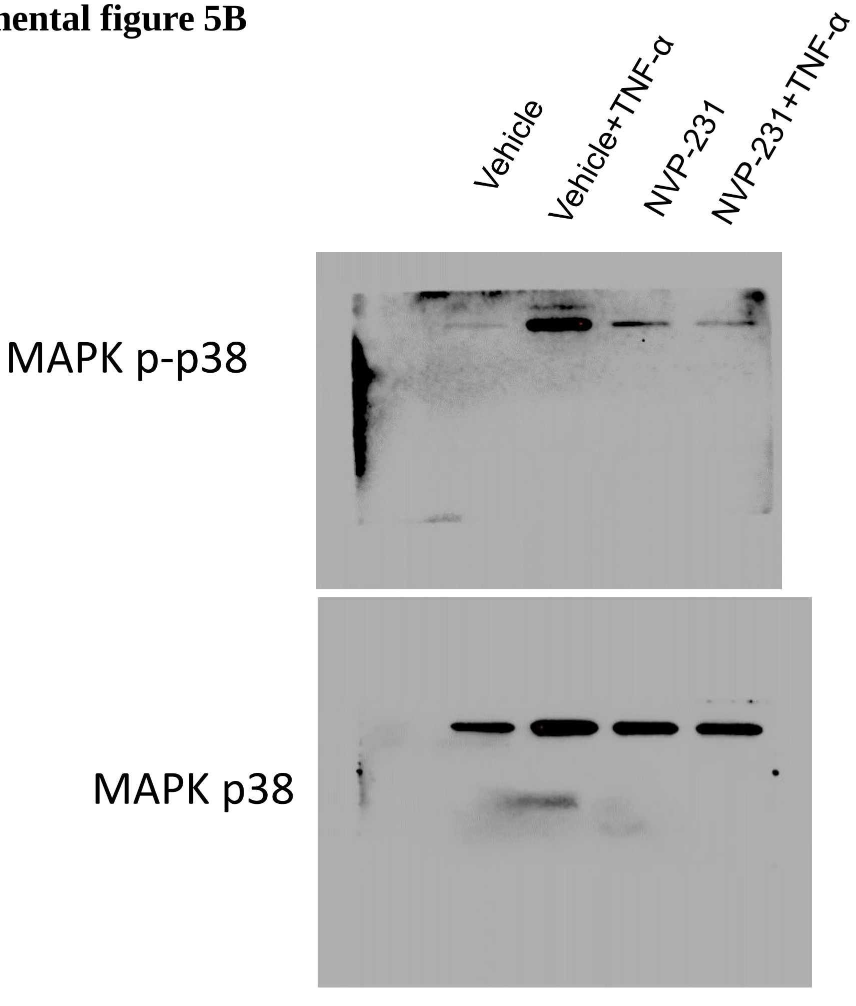

Supplemental figure 5C

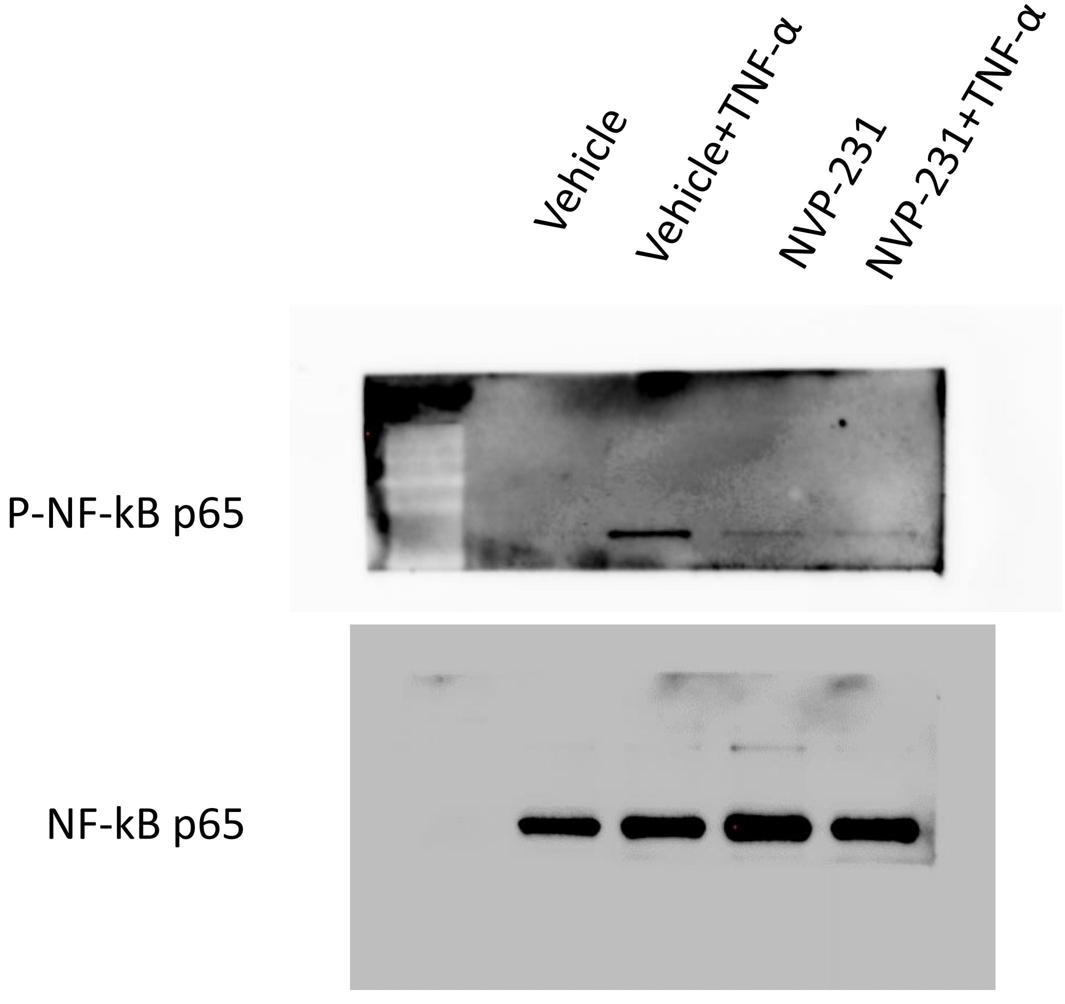

Supplement: Supplementary file 1 — Supplementary Information. [file 41598_2021_87795_MOESM1_ESM.pdf]
